# Temporal Dynamics of Fungal Communities in Alkali-Treated Round Bamboo Deterioration under Natural Weathering

**DOI:** 10.3390/microorganisms12050858

**Published:** 2024-04-25

**Authors:** Shuaibo Han, Xiaojiao An, Xiaolong He, Xin Ren, John Sichone, Xinxing Wu, Yan Zhang, Hui Wang, Fangli Sun

**Affiliations:** 1 National Engineering & Technology Research Center of Wood-Based Resources Comprehensive Utilization, School of Chemical and Materials Engineering, Zhejiang A&F University, Hangzhou 311300, China; axj624@stu.zafu.edu.cn (X.A.); 2022604051005@stu.zafu.edu.cn (X.H.); rx@stu.zafu.edu.cn (X.R.); johnsichone450@gmail.com (J.S.); xinxingwu@zafu.edu.cn (X.W.); zhangy@iccas.ac.cn (Y.Z.); wanghui@zafu.edu.cn (H.W.); 2 Microbes and Insects Control Institute of Bio-Based Materials, Zhejiang A&F University, Hangzhou 311300, China

**Keywords:** alkali treatment, bamboo deterioration, fungal community

## Abstract

Microbes naturally inhabit bamboo-based materials in outdoor environments, sequentially contributing to their deterioration. Fungi play a significant role in deterioration, especially in environments with abundant water and favorable temperatures. Alkali treatment is often employed in the pretreatment of round bamboo to change its natural elastic and aesthetic behaviors. However, little research has investigated the structure and dynamics of fungal communities on alkali-treated round bamboo during natural deterioration. In this work, high-throughput sequencing and multiple characterization methods were used to disclose the fungal community succession and characteristic alterations of alkali-treated round bamboo in both roofed and unroofed habitats throughout a 13-week deterioration period. In total, 192 fungal amplicon sequence variants (ASVs) from six phyla were identified. The fungal community richness of roofed bamboo samples declined, whereas that of unroofed bamboo samples increased during deterioration. The phyla Ascomycota and Basidiomycota exhibited dominance during the entire deterioration process in two distinct environments, and the relative abundance of them combined was more than 99%. A distinct shift in fungal communities from Basidiomycota dominant in the early stage to Ascomycota dominant in the late stage was observed, which may be attributed to the increase of moisture and temperature during succession and the effect of alkali treatment. Among all environmental factors, temperature contributed most to the variation in the fungal community. The surface of round bamboo underwent continuous destruction from fungi and environmental factors. The total amount of cell wall components in bamboo epidermis in both roofed and unroofed conditions presented a descending trend. The content of hemicellulose declined sharply by 8.3% and 11.1% under roofed and unroofed environments after 9 weeks of deterioration. In addition, the contact angle was reduced throughout the deterioration process in both roofed and unroofed samples, which might be attributed to wax layer removal and lignin degradation. This study provides theoretical support for the protection of round bamboo under natural weathering.

## 1. Introduction

Bamboo is an evergreen, flowering woody grass belonging to the family Poaceae and subfamily Bambusoideae. Due to its wide distribution, rapid growth rate, and superior mechanical properties, bamboo is widely used in various structural engineering applications, such as construction, the automotive industry, and logistics. In comparison with traditional structural building materials, such as steel and concrete, bamboo exhibits an outstanding carbon sequestration capacity, which makes bamboo products an environmentally friendly and sustainable alternative to conventional structural building products [1]. Round bamboo is the original form of natural bamboo, consisting of a hollow cylindrical culm divided into sections by nodes. The external wall of round bamboo is composed of bamboo epidermis, cortex, ground tissue, and vascular bundles from exterior to interior. The epidermis is the outer layer of the culm, composed of elongated cells, short cork and silica cells, and the stomata [2]. In contrast to modern bamboo engineering materials, which crush bamboo culms into small units, round bamboo culms maintain the natural texture, shape, and mechanical structure of raw bamboo materials [3]. Owing to its attractive appearance, high strength, and bending modulus, round bamboo has served as the most historically used form of bamboo material and has gained growing attention in building, decorating, and furnishing [4,5].

Nevertheless, mildew and decay caused by various microbes pose the primary threat to the appearance and durability of round bamboo [6]. The fresh round bamboo culms contain a high concentration of organic nutrients, including 2.2–5.18% starch, 2% soluble sugar, 2.18–3.55% fat and wax, 1.5–6% protein, and over 30% water [7]. Among them, starch and free sugars generally act as the nutrient reserves of plants, but in the meantime, they are the main food sources of fungi [8]. Therefore, when round bamboo is exposed to a moist environment, especially outdoors, during storage, processing, and usage, mold and decay fungi colonize it vigorously and affect its appearance and aesthetic quality drastically. 

Mildew is the result of mold growth, and susceptibility to mold development depends on both the type of bamboo used and the atmosphere in which it is grown [9]. Molds tend to vegetate at the cross-ends and on the surface of bamboo culms in a humid environment as they require high relative humidity above 70% [10]. The majority of bamboo mold fungi are Ascomycetes and are classified into many asexually reproducing genera, including *Alternaria*, *Aspergillus*, *Fusarium*, *Globisporium*, *Penicillium*, and *Trichoderma* [11]. The pigment secreted by fungal mycelium will leave gray, brown, or blue mildew on the bamboo surface, resulting in a significant depth of staining that cannot be removed by bleach treatment [12]. Discoloration caused by mold fungi has a substantial impact on the aesthetic aspects of round bamboo, severely limiting its application and resulting in massive economic losses and waste of bamboo resources. Furthermore, when mold spores are present in high concentrations in the air, they can induce allergic responses, asthma attacks, infections, and other respiratory issues in humans [13]. 

Damage caused by decay fungi is another serious issue while storing and using round bamboo. These fungi are categorized as brown-rot (BR), white-rot (WR), and soft-rot (SR) types based on their function. Contrary to SR and BR, numerous WR-inducing fungi can metabolize all three principal polymers found in bamboo, which are lignin, cellulose, and hemicelluloses. The SR-causing fungi selectively break down cellulose and hemicelluloses and make small modifications to lignin, whereas the BR fungi exclusively focus on cellulose and hemicellulose. The occurrence of SR and WR fungi is more prevalent on bamboo substrates than that of BR fungi [14,15]. Factors such as environmental temperature and humidity, moisture content, and nutrient status in the culm determine the severity of attack and colonization by the microbes [16]. Okahisa et al. (2006) investigated seasonal fluctuations in sugar levels in immature culms and discovered that a high glucose content promotes fungal breakdown [8]. Young culms are more vulnerable to BR, resulting in larger mass losses compared to WR fungi [14,17]. This might be attributed to increasing local lignin concentrations in mature culms, which physically restrict hyphae development and reduce the local equilibrium moisture content [18]. It has been reported that anatomical parts (piths) and ultrastructural features (micro-fibril angle) were able to determine hypha growth direction and branching mechanisms [19]. These barriers can only postpone, rather than prevent, fungus from damaging the substrate [18]. 

It has become evident that mold and decay in the natural environment do not result from individual strains and taxa of microbes acting alone but from the emergent properties of microbial communities as a whole [20]. Therefore, unveiling the dynamic changes of the fungal community is a critical step to understanding the microbial mechanism of the biological deterioration of round bamboo. Temporal variation refers to the process by which the structure of a biological community evolves over time. Such temporal variations in plant [21] and animal [22] communities have been intensively investigated to establish links between community stability and biodiversity as well as forecast community responses to disturbances [23]. Microorganisms are ubiquitous and vital to ecosystems, yet little is known about their temporal dynamics, which may be attributed to the practical difficulties of measuring microbial species and the lack of a clear definition of species [24,25]. As to the biological deterioration of round bamboo, to our knowledge, the temporal dynamics of the fungal community during this process remain largely unknown. Zhou and Hyde proposed that seasonality has an effect on fungal succession and that the fungal community on bamboo can be categorized into early, middle, and later stage colonizers based on observing fruit bodies in the laboratory via culturing fungi of bamboo culms on medium [26]. However, because of the “selective” effect of any cultural conditions, the culture-dependent method was impossible to capture the whole scenario of the microbial community [27]. While assessing the temporal dynamics of microorganisms is a difficult task, advancements in this area have been facilitated by culture-independent techniques such as high-throughput sequencing, which can identify the vast majority of microbial taxa found in a given sample [28]. 

In our previous research, we investigated the succession of fungal communities on fresh round bamboo during the natural deterioration of three months [29]. Nevertheless, alkali-treated round bamboo was more commonly used than fresh round bamboo in the daily lives of individuals. Round bamboo culms are initially green in color but will gradually turn yellow as they are used. Alkali treatments are often employed for the treatment of fresh round bamboo to alter its natural color to yellow in a relatively short time. Moreover, the current bamboo modification procedures are unable to penetrate the inside of the bamboo due to the presence of a silicon wax layer on its surface [30]. Alkali treatment with proper concentration was able to remove the weaker matrix of the bamboo layer and impose little effect on the fibers [31]. Therefore, alkali treatments were also performed to increase the permeability of round bamboo, resulting in ideal conditions for further modifications [30]. Additionally, in contrast to some current bamboo modification techniques, such as acid-base treatment, freeze-drying, and microwave blasting, alkali treatment offers several advantages, including simple processing, reduced expenses, improved production efficiency, and increased competitiveness [32]. However, alkali-treated round bamboo was prone to mold and decay as well, and the temporal dynamics of the fungal community are still unknown. 

To address this gap in our knowledge, we explored the dynamic changes of fungal communities on alkali-treated round bamboo using high-throughput sequencing and multivariate data analysis. Meanwhile, the morphology, chemical components, and other features of alkali-treated round bamboo in different degradation stages were investigated using various characterization methods, such as scanning electron spectroscopy (SEM), X-ray photoelectron spectroscopy (XPS), high-performance liquid chromatography (HPLC), and contact angle analysis. This study aimed to address the following questions: (1) What is the fungal diversity and community composition during the biodeterioration of alkali-treated bamboo under natural weathering? (2) How about the temporal variations of fungal diversity and community during this process, and what is the relationship between fungal community variations and round bamboo characteristics? (3) What are the mechanisms underlying the community assembly of fungi during the biodeterioration of alkali-treated round bamboo? The study provides a foundation for further studies of alkali-treated round bamboo deterioration mechanisms and facilitates the protection of round bamboo in outdoor environments.

## 2. Materials and Methods

### 2.1. Study Site and Sampling 

The sampling site of this study was on the campus of Zhejiang A&F University, Hangzhou, Zhejiang province (119°44′ E, 30°15′ N). Being a typical region in southern China, this district also undergoes a period of rainfall in the middle of each year. The Meiyu season, also known as the rainy period, often starts in early June and ends in mid-July [33], and the precipitation during this period is mostly stable and continuous [34]. The optimal temperature and elevated humidity during this season promote the growth of mold and decay, affecting not only textiles but also wood products. 

**Figure 1:**
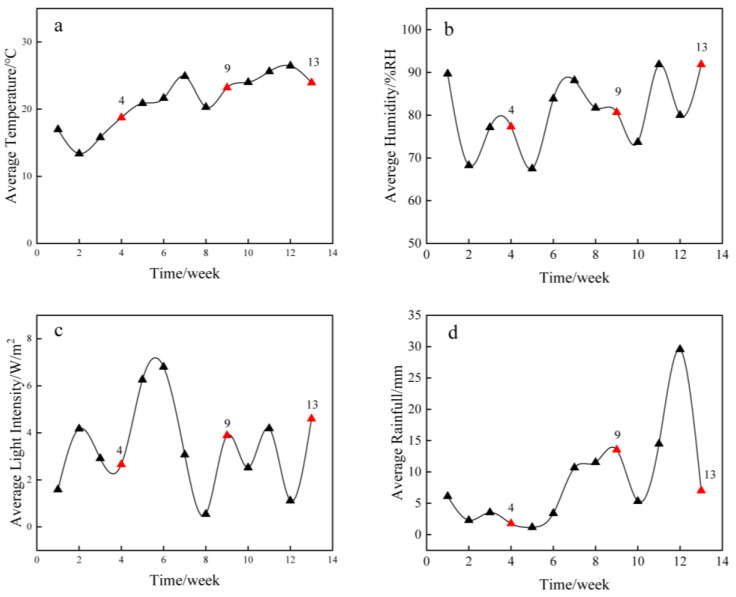
Temperature (**a**), humidity (**b**), light intensity (**c**), and rainfall (**d**) variations during the deterioration process. The red triangle indicates the sampling time.

Four-year-old bamboo (*Phyllostachys iridescens*) was collected from the bamboo forest in Anji County, Zhejiang, China, in 2021 (119°14′ E, 30°53′ N). Bamboo culms with a diameter of 10 cm and above the bottom up to 2–4 m were processed into 30-cm-long round bamboo segments. The alkali solution was prepared using 2.5 mol/L NaOH and 0.4 mol/L Na_2_CO_3_ and mixed in a ratio of 1:1 (*v*/*v*). A relatively small amount of Na_2_CO_3_ was used as an adjuvant to improve the permeability of round bamboo. The round bamboo samples were immersed in an alkali aqueous solution at 90 °C for two hours. After the treatment, the samples were air-dried for 3 days. Subsequently, the culms were sterilized with an 80% alcoholic solution. Two biologically hazardous conditions for round bamboo segments C3.1 (exterior, above-ground, roofed) and C3.2 (exterior, above-ground, unroofed) were set based on Chinese National Standard GB/T 27651-2011 [35]. As shown in Figure S1, round bamboo segments were placed on the rack with the bottom 20 cm above ground. Two groups, roofed (H) and unroofed (HY), were set to simulate C3.1 and C3.2 conditions, respectively. HY groups were directly exposed to the rainfall, whereas H groups were covered with plastic sheeting to avoid exposure to the rain. Bamboo samples were collected at the initial (week_4), mid-term (week_9), and later stages (week_13) of the Meiyu season, and each stage was sampled using three biological replicates. A total of 18 round bamboo samples were obtained, including 9 roofed bamboo samples and 9 unroofed bamboo samples. Each of the round bamboo samples was sampled once for DNA extraction and characterization analysis. To avoid fungal contamination from both ends, a 1-mm-deep central zone of bamboo segments was chosen for DNA extraction in each sample. The environmental data (i.e., temperature, humidity, illumination, and rainfall) were provided by the Lin’an Meteorological Bureau (Figure 1). 

### 2.2. Fungal Community Analysis 

#### 2.2.1. DNA Extraction and Sequencing

The extraction of total DNA from bamboo samples was performed using the TGuide S96 Magnetic DNA Kit (Tiangen Biotech (Beijing) Co., Ltd., Beijing, China) under the manufacturer’s instructions. The Qubit dsDNA HS Assay Kit and Qubit 4.0 Fluorometer (both manufactured by Thermo Fisher Scientific, Waltham, MA, USA) were employed to quantify the concentration and purity of the extracted DNA. ITS1 (5′-CTTGGTCATTTAGGAAGTAA-3′) and ITS4 (5′-TCCTCCGCTTATTGATATGC-3′) were employed as full-length primers specific to fungi [36]. Unique barcode sequences were added to both the forward and reverse primers to facilitate sequencing numerous samples simultaneously.

The polymerase chain reaction (PCR) was conducted utilizing the KOD One PCR Master Mix (TOYOBOLife Science, Osaka, Japan) under the specified conditions: initial denaturation at 95 °C for 2 min; 25 cycles at 98 °C for 10 s, 55 °C for 30 s, and 72 °C for 90 s; and final extension at 72 °C for 10 min. Purification of the PCR product was performed using Agencourt AMPure XP Beads (Beckman Coulter, Indianapolis, IN, USA), and quantification was performed using a Qubit dsDNA HS Assay Kit (Invitrogen, Thermo Fisher Scientific, Waltham, MA, USA) [37]. Following individual quantification, the amplicons were pooled in equal amounts. Utilizing SMRTbell Express Template Prep Kit 2.0 (Pacific Biosciences, Menlo Park, CA, USA), SMRTbell libraries were generated from amplified DNA. The amplicon libraries were sequenced on a single PacBio Sequel II 8M cell using the Sequel II Sequencing Kit 2.0 [38]. 

#### 2.2.2. Sequence Data Processing 

The bioinformatics analysis in this study was performed using BMK Cloud (Biomarker Technologies Co., Ltd., Beijing, China). SMRT Link software version 8.0 (Pacific Biosciences, Menlo Park, CA, USA) was used to process raw sequencing reads into circular consensus sequencing (CCS). The CCS sequences were matched to the respective samples using their barcodes, facilitated by the Lima software version 1.7.0 (Pacific Biosciences, Menlo Park, CA, USA). The Cut adapt quality control method (version 2.7) was employed to eliminate CCS reads lacking primers and reads above the length range of 1200–1650 bp. Clean reads were acquired by recognizing and eliminating chimeric sequences with the UCHIME method [39]. The DADA2 method in QIIME 2 [40] (v2020.06) was applied to de-noise sequences, generating amplicon sequence variants (ASVs), and ASVs with a redundancy level below 0.005% were removed. The ASVs were taxonomically annotated using the UNITE version 7.1 database (https://unite.ut.ee/, Tartu, Estonia). Rarefaction curves were used for assessing sequencing depth. 

#### 2.2.3. Statistical Analysis 

The QIIME2 and R Studio v1.2.5033 software (RStudio, Inc.: Boston, MA, USA) were used to calculate and present the alpha diversity, which includes the ACE, Chao1, Shannon, and Simpson indexes [41]. QIIME 2 was also used to analyze the beta diversity, including the unweighted pair-group method with arithmetic means (UPGMA), heat maps, and principal coordinate analysis (PCoA). The redundancy analysis (RDA) was performed in R with the package ‘vegan’ to explore the differences in the microbiome among different factors. The FUNGuild database was employed to allocate fungal communities to functional guilds. (e.g., phytopathogenic, parasitic, and saprophytic). 

### 2.3. Characteristics of Bamboo

#### 2.3.1. Chemical Composition Determination

The outer layer of bamboo culms, with a thickness of 1 mm, was removed and crushed into a fine powder with a particle size of 80 mesh. The bamboo powder was dried at three different temperatures: 60 °C for two hours, 80 °C for two hours, and 105 °C until it reached a constant weight. The extractive content of the bamboo sample was determined using a Soxhlet extractor (Jiangsu Huida Medical Instruments Co., Ltd., Yancheng, China) and an ethanol–benzene solution for 8 h. Benzene and ethanol are mixed in a volumetric ratio of 2:1. The cellulose, xylan, arabinan, acid-soluble lignin (ASL), acid-insoluble lignin (AIL), and ash contents of bamboo samples were determined using a procedure proposed by the National Renewable Energy Laboratory (NREL, Golden, CO, USA). Moreover, 0.3 g of each sample was steeped in 72% (*w*/*w*) H_2_SO_4_ at 30 °C for 1 h, then hydrolyzed in 4% (*w*/*w*) H_2_SO_4_ at 121 °C for another hour. The acid-hydrolysis solution was subsequently analyzed using HPLC to determine its carbohydrate content. The cellulose content of the bamboo samples was represented by glucose. The hemicellulose content was represented by the sums of xylan and arabinan. Moreover, 2 g of each sample was weighted and burned at 575 °C for 12 h to assess the ash content. The burnt residue’s dry weight, divided by the bamboo powder sample, revealed the ash content [42].

#### 2.3.2. XPS Analysis

The finely ground bamboo epidermis, obtained by scraping, was analyzed using a Thermo Scientific K-Alpha XPS system (Thermo Fisher Scientific, Cleveland, OH, USA). The device utilized a monochromatic Al KαX-ray source with an energy of 1486.6 eV. The survey spectra were collected using a pass energy of 150 eV and a binding energy (BE) range of 0 to 1350 eV. The peaks were inspected, and high-resolution scans were acquired using a pass energy of 50 eV. The spectra were acquired using an analysis area with a diameter of 400 nm. The peaks of all samples were calibrated using the binding energy (BE) of C1s graphitic carbon, which is specified at 284.6 eV, to correct the charge effects. The C and O peaks were deconvoluted into subcomponents using the Lorentzian-Gaussian distribution following background subtraction based on the Shirley method, utilizing the XPS peak 4.1 software package. 

#### 2.3.3. SEM Observation

A scanning electron microscope (SEM) (TM3030, HITACHI, Tokyo, Japan) was used to study the alterations on the bamboo epidermis. Bamboo culms were cut into little pieces. The epidermis was then collected and carbon taped to the metal sample table. SEM scanning was performed under vacuum at 15 kV voltages following a 10 nm gold film coating. 

#### 2.3.4. Contact Angle Measurements

Contact angle analysis was performed to determine the hydrophobic surface of bamboo using an OCA50AF contact angle measurement tool (Dataphysics, Filderstadt, Germany). The volume of the deionized water droplet was set to 5 μL, and the test time was set to 5 s. 

## 3. Results and Discussion

### 3.1. Variations in the Diversity of the Fungal Community 

High-throughput sequencing was adopted to evaluate the fungal communities present on bamboo surfaces at various stages. A total of 219,396 quality sequences were obtained from bamboo samples at three different stages, which were assigned to 192 amplicon sequence variants (ASVs) (Figure S2). Shannon index curves, in addition to rarefaction curves, were used to evaluate whether the sequencing data amount was enough to cover all the fungal species and to reflect species richness in these samples. As presented in Figures S3 and S4, the rarefaction curves and Shannon curves tended to be flat and stable, indicating that the sequencing data from this research adequately represented the whole fungal diversity.

#### 3.1.1. Alpha Diversity Analysis

**Figure 2:**
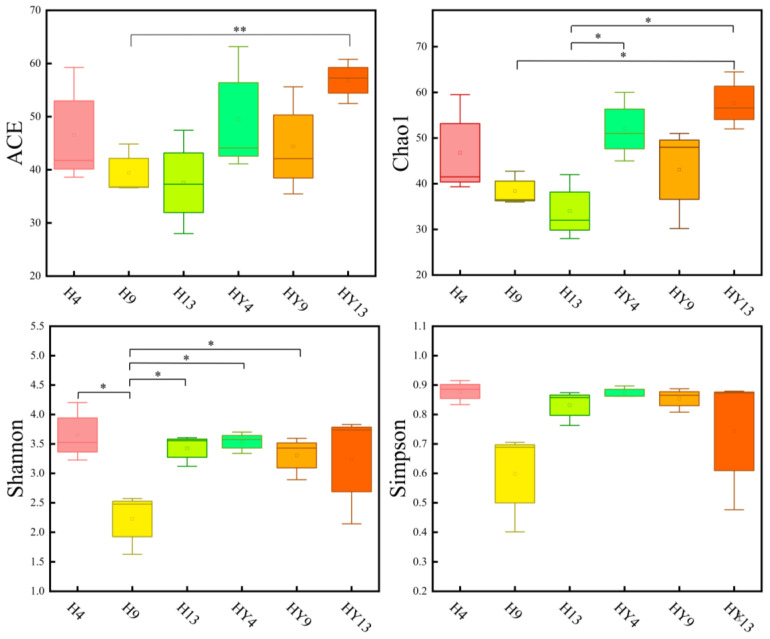
Fungal richness (ACE and Chao1) and diversity (Shannon and Simpson) indexes during alkali-treated round bamboo deterioration. Asterisks (* and **) indicate significant differences at *p* < 0.05 and *p* < 0.01 probability levels, respectively.

Many indexes, including Chao1, ACE, Shannon, and Simpson, can be used to reflect the Alpha diversity. Community richness is shown by Chao1 and ACE, while community evenness is presented by the Shannon and Simpson indexes. In this study, the Chao1 and ACE indexes of the alkali-treated roofed round bamboo after exposure for four (H4), nine (H9), and thirteen (H13) weeks showed a decreasing trend during deterioration, whereas those of the unroofed groups (HY4, HY9, and HY13) decreased and then increased after week_9 (Figure 2). Moreover, H13 exhibited the lowest values of the fungal ACE index and Chao 1 index, with 37.57 and 34, respectively. The highest values of bamboo fungal ACE index and Chao 1 index were observed in HY13, with 56.84 and 57.7, respectively. This result was different from our previous research on fresh round bamboo, which showed that community richness presented increasing and declining trends in roofed and unroofed groups, respectively, during deterioration [29]. One of the reasons is that the immersion of an alkaline solution was able to remove some nutrient sources for fungi, such as soluble starch and sugars, forming a barren and harsh environment for microorganisms. In addition, because most fungi thrive at slightly acidic pH values of 5.0–6.0 [43], the high pH micro-environment established by the alkali treatment can inhibit the growth of fungi. Liu et al. [44] found that the Chao and ACE indexes of the fungal community of saline-alkali soil were significantly lower than those of untreated control samples. As to the unroofed groups, constant flushing by rainfall may alter the pH of the bamboo surface, leading to an increase in fungal community richness. The Shannon and Simpson index of roofed groups decreased and then increased from week_4 to week_13. As to the unroofed groups, the Shannon and Simpson index tended to be stable during the deterioration process. Among all groups, H9 had the lowest Shannon and Simpson index, indicating low diversity in a fungal community. Unexpectedly, H4 presents the highest Shannon and Simpson index with 3.54 and 0.87, respectively. 

**Figure 3:**
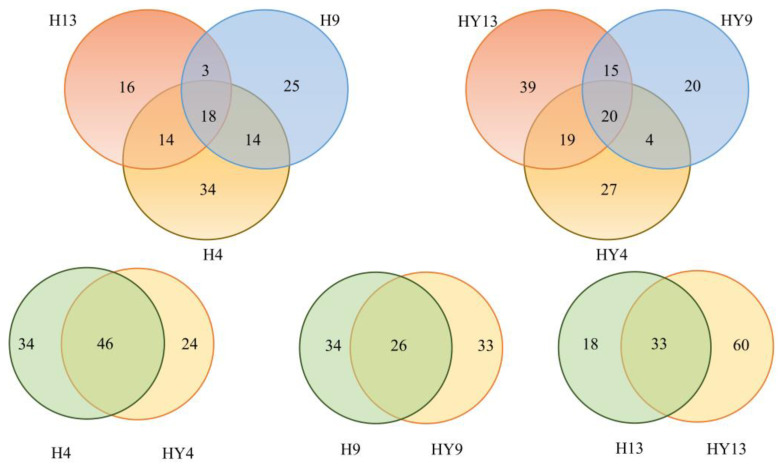
Venn diagram exhibiting the common or unique ASVs among different groups.

Venn diagrams provide a visual representation of the similarities and overlaps between different groups. In Figure 3, it can be observed that the H4, H9, and H13 groups shared a total of 18 ASVs, while having 34, 25, and 16 unique ASVs, respectively. Similarly, the HY4, HY9, and HY13 groups shared 20 ASVs, with 27, 20, and 39 unique ASVs, respectively. The overlapping ASVs between H4 and HY4, H9 and HY9, and H13 and HY13 were 46, 26, and 33, respectively. 

#### 3.1.2. Beta Diversity Analysis 

**Figure 4:**
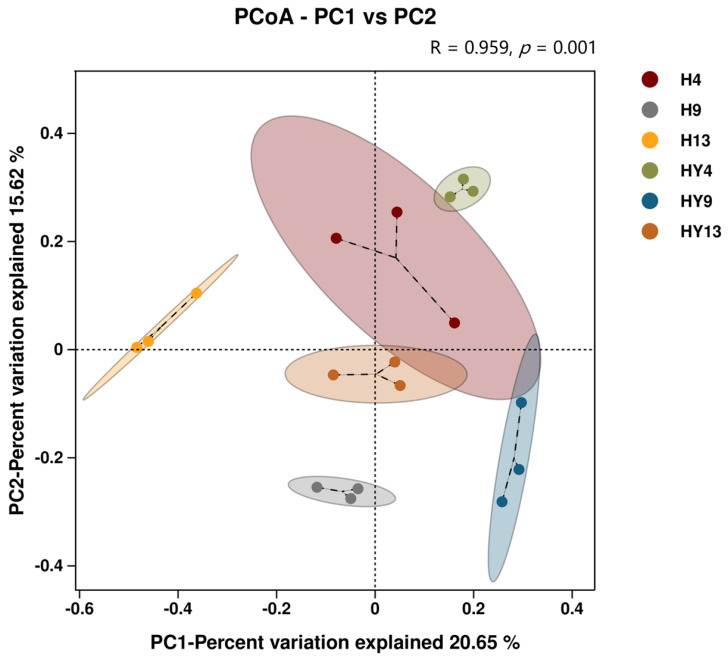
Principle coordinate analysis (PCoA) plot based on the binary Jaccard distance algorithm at the ASV level. Each point in the plot represents a sample, and samples from the same group are represented in the same color.

Principal coordinate analysis (PCoA) was performed to evaluate overall differences in the structures of fungal communities based on binary Jaccard distances. As shown in Figure 4, the difference in contribution rate of microbial community structure in the first two principal components (PC1 and PC2) was 20.65% and 15.62%, respectively. The distribution of different samples on the PCoA plot was separated distinctly. The results of the ANOSIM test (R = 0.959, *p* = 0.001) revealed that the difference between groups was much greater than that within groups. UPGMA dendrograms of the fungal community showed similar results as the PCoA plot (Figure S5). Furthermore, samples with the same collection time frequently cluster together. This result indicates that, compared with different rainfall environments, different deterioration times make a greater contribution to the variation of fungal communities and thus have a greater impact on the succession of fungal communities on round bamboo. This result was in accordance with our previous research on fresh round bamboo [29]. In addition, samples of roofed groups (i.e., H9-1, H9-2, and H9-3) were clustered into one branch, while samples of unroofed groups (i.e., HY9-1, HY9-2, and HY9-3) were clustered into another branch in UPGMA dendrograms, which illustrated that rainfall, to some extent, contributes to this divergence. Meanwhile, H13 and HY13 groups gathered together and formed a distinct branch from other groups, which indicated that the fungal community varied drastically during the late stage of the alkali-treated bamboo deterioration process. 

### 3.2. Microbial Community Composition 

**Figure 5:**
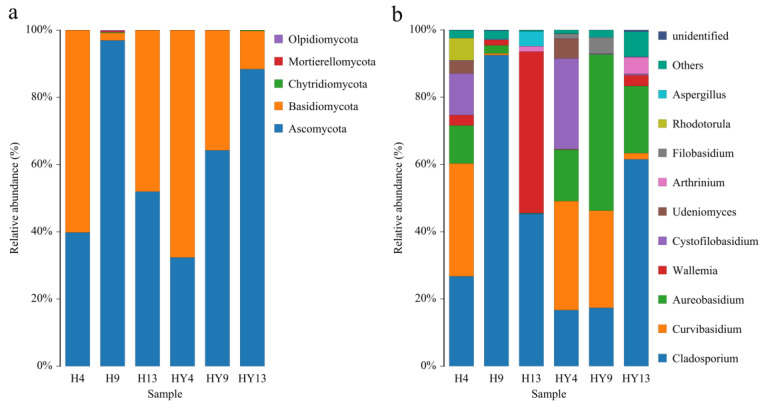
Histograms of the relative abundance of fungal communities in bamboo outdoor deterioration at the phylum (**a**) and genus (**b**) levels.

At the phylum level, Ascomycota and Basidiomycota were identified as the dominant phyla throughout the deterioration period (Figure 5a). This result was consistent with the previous studies, where they were the main fungal groups on bamboo [45]. In roofed groups, the relative abundance of Basidiomycota was 60.2%, 2.24%, and 48.04% in H4, H9, and H13 groups, respectively. In unroofed groups, the relative abundance of Basidiomycota was 67.63%, 35.75%, and 11.37% in the HY4, HY9, and HY13 groups, respectively. Ascomycota was another dominant phylum in the fungal communities, comprising 39.80%, 96.95%, and 51.96% in the H4, H9, and H13 groups, respectively. Whereas the relative abundance of Ascomycota in unroofed groups was 32.37%, 64.24%, and 88.39%, respectively. One of the similarities between the fungal community composition of roofed and unroofed groups is that Basidiomycota, instead of Ascomycota, was dominant at the early stage, while Ascomycota gradually turned into the dominant phylum at the late stage of deterioration. Generally, it is believed that fungi belonging to the phylum Ascomycota tend to grow and spread at an earlier stage of decay compared to fungi in the phylum Basidiomycota, which result from the varying abilities of Basidiomycetes to break down plant biopolymers [46]. For instance, the majority of white rot fungi, which are assumed to be the main decomposers of resistant plant biopolymers like lignin that have greater concentration in the late stage of decay, belong to the phylum Basidiomycota [47]. However, the results from this study presented an opposite shift in the fungal community during the decomposition process of alkali-treated round bamboo, which may be attributed to several reasons. The alkali treatment was able to remove the compact silicon wax layer and reduce the content of soluble sugar and starch on the round bamboo surface, resulting in the exposure of lignin and cellulose, which are the primary carbon sources of some Basidiomycota fungi. Additionally, priority effects may also play an important role in fungal community assembly during the natural deterioration process of round bamboo. The priority effect is the phenomenon that the outcome of species interaction often depends on the temporal sequence of community assembly [48]. In other words, the order and timing of species arrivals can have an impact on community development [49]. The earlier arrival species alter resources or environmental conditions, which impact species that arrive later, affecting their ability to establish themselves in the community [50]. As the earlier arrival colonizers, many Basidiomycota fungi were able to degrade lignin organic acid and increase the accessibility of cellulose and hemicellulose [51], providing a favorable environment for the flourishing of Ascomycota [52]. 

At the genus level, our results showed that the representatives of the genera *Arthrinium*, *Aspergillus*, *Aureobasidium*, *Cladosporium*, *Curvibasidium*, *Cystofilobasidium*, *Filobasidium*, *Rhodotorula*, *Udeniomyces*, and *Wallemia* were the dominant taxa (Figure 5b). *Cladosporium* dominated the entire deterioration process in both roofed and unroofed groups, especially in H9 (92.52%) and HY13 (61.53%). This result was consistent with our previous research on fresh round bamboo [29]. *Cladosporium* is a large genus of dematiaceous hyphomycetes fungus and has a broad lifestyle due to its strong adaptation to different environments [53]. *Cladosporium* spp. is widely distributed in various bamboo materials, such as moso bamboo seeds [54], decayed leaves of bamboo [53], and dead bamboo culms [55]. *Curvibasidium* was the predominant fungal genus in H4 (33.55%), HY4 (32.42%), and HY9 (38.91%). The genus was a yeast-like fungus and was proposed by Sampaio et al. [56]. The species of the genus *Curvibasidium* were widely distributed in various environments, e.g., white grapevine [57], litter of temperate forests [58], and marine sediments [59]. *Curvibasidium pallidicorallinum* has been shown to generate polygalacturonase, which might potentially improve the breakdown of berries’ cell walls during fermentation [60]. It’s worth noting that *Cystofilobasidium*, which was one of the dominant genera at the initial stage in both roofed and unroofed environments, almost disappeared after 13 weeks of deterioration. The genus *Cystofilobasidium* was proposed by Oberwinkler et al. in 1983 to accommodate two teliospore-forming yeast species, i.e., *Cystofilobasidium bisporidii* and *Cystofilobasidium capitatum*, which were previously classified as members of the genus *Rhodosporidium* [61]. *Cystofilobasidium* species have been observed inhabiting a wide range of habitats, including lake water [62], forest soil [63], and arctic glaciers [64]. Interestingly, recent research has revealed that *Cystofilobasidium* is one of the primary taxa in the gut microbiomes of giant pandas, which mainly feed on bamboo [65,66,67]. However, to the best of our knowledge, there have been no reported cases of *Cystofilobasidium* inhabiting bamboo surfaces, and our findings have addressed this gap. 

### 3.3. The Correlation between Environmental Factors and Fungal Communities

**Figure 6:**
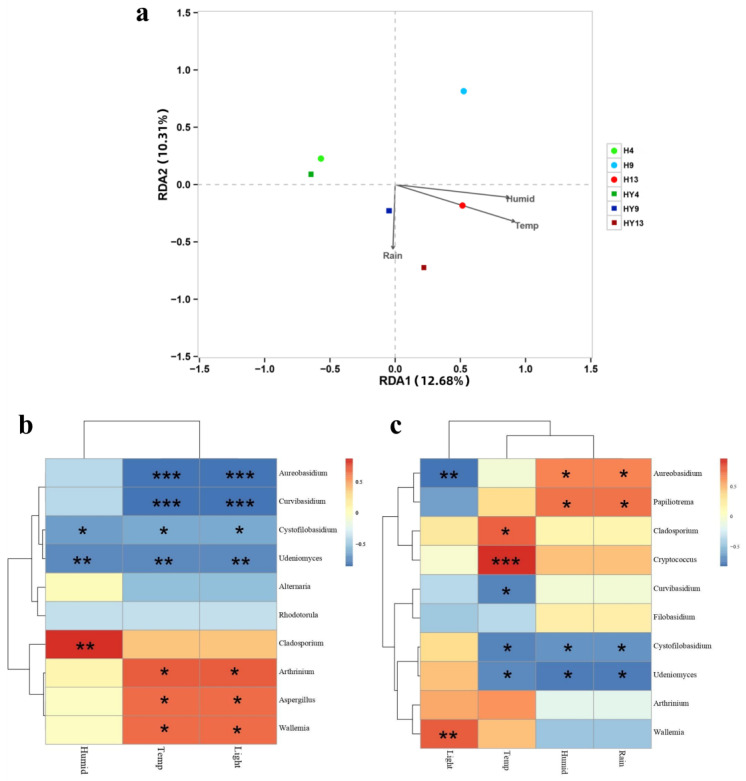
Redundancy analysis (RDA) plot representing the relationship between fungal community composition and environmental factors (**a**). Heat map of the correlation between environmental factors and fungal communities at the genus level of the roofed (**b**) and unroofed groups (**c**). The colors in the heat maps indicate the Spearman correlation coefficient r, which ranges from −1 to 1; r < 0, negative correlation (blue); r > 0, positive correlation (red). Asterisks represent significance levels: * *p* < 0.05, ** *p* < 0.01, and *** *p* < 0.001. Humid: humidity; Temp: temperature; Rain: rainfall.

Redundancy analysis was employed to reveal the relationship between environmental factors and fungal communities (Figure 6a). The results showed that out of the four environmental factors gathered, three factors were statistically significant in elucidating the variability of fungal community structure (*p* < 0.01). However, they only account for 22.99% of the overall variability. Axis 1 accounted for 12.68% of the variance, whereas axis 2 accounted for 10.31%. Temperature was the primary factor influencing the variance in bamboo fungal community composition, followed by humidity and rainfall. Previous research also revealed that the temperature on the surface of decomposing needle litter from *Pinus densiflora* had a significant role in the dynamics of fungal communities [68]. 

Correlation analysis shows that, in roofed groups, genera *Arthrinium*, *Aspergillus*, and *Wallemia* were positively correlated with temperature and light, and *Cladosporium* was positively correlated with humidity. Meanwhile, *Aureobasidium* and *Curvibasidium* were negatively correlated with temperature and light, and *Cystofilobasidium* and *Udeniomyces* were negatively correlated with these three environmental factors. Among unroofed groups, the correlation analysis identified that *Aureobasidium* and Papililotrema were positively correlated with humidity and rainfall, while *Aureobasidium* also showed a negative correlation with light. The genera *Cladosporium* and *Cryptococcus* had a positive correlation with temperature. Moreover, *Cystofilobasidium* and *Udeniomyces* were negatively correlated with temperature, humidity, and rainfall. Notably, as the yeast-like genera, *Curvibasidium* and *Cystofilobasidium* were prevalent at the initial stage (week_4), accounting for 33.55% and 12.38% of total taxa in the H4 group, respectively, and 32.42% and 26.99% in the HY4 group, respectively. However, those two genera almost disappeared after 13 weeks of deterioration. As mentioned above, both *Curvibasidium* and *Cystofilobasidium* were negatively correlated with temperature. It has been reported that many *Curvibasidium* and *Cystofilobasidium* species thrive at relatively low temperatures. Bai et al. [69] found that *Curvibasidium* sp. Y231 showed optimal growth at 20 °C. The maximum growth temperature of *Cystofilobasidium josepaulonis* is 28 °C, and the optimal growth temperature is 17–20 °C [70]. Therefore, we speculated that rising temperatures were the main reason that led to the almost disappearance of *Curvibasidium* and *Cystofilobasidium* during 13 weeks of deterioration. 

### 3.4. Function of Fungi 

**Figure 7:**
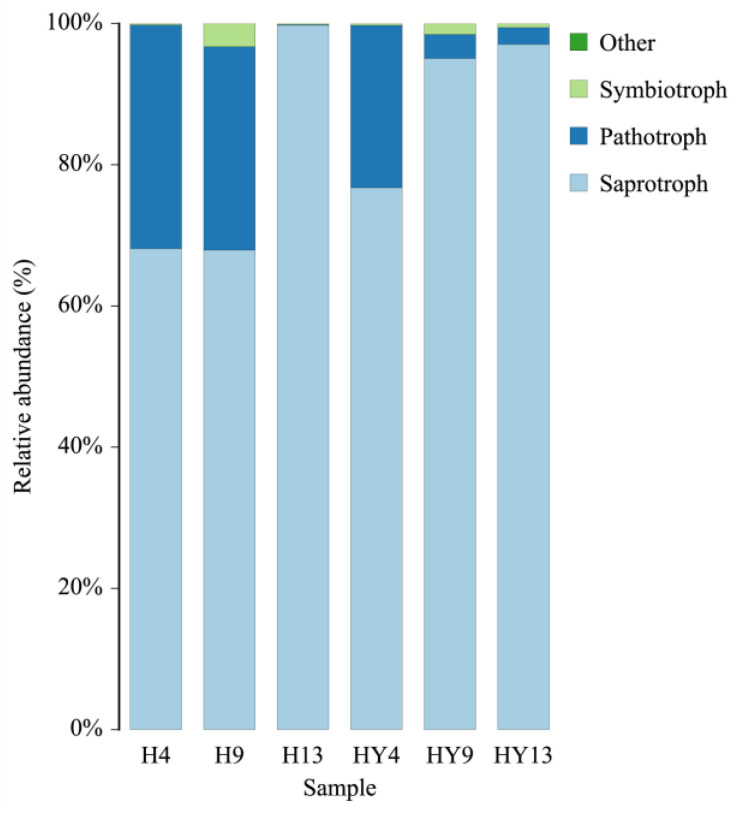
Relative abundance of fungal functional groups (guilds) at trophic level.

The FUNGuild database was utilized for fungal function prediction. The abundance of saprotrophic modes was the highest across all groups, as illustrated in Figure 7. Saprotrophs are fungal organisms that derive energy from the decomposition of organic matter, including leaf detritus and wood. They are crucial in nutrient cycling and organic matter breakdown and play a significant role in maintaining soil health. Nevertheless, saprotrophic organisms presented on bamboo surfaces may weaken their structural integrity and adversely affect their mechanical properties. Moreover, the relative abundance of saprotrophs in roofed groups was lower than that of unroofed groups. This result was different from our previous study on fresh round bamboo, which showed an opposite appearance [29]. Tervonen et al. [71] revealed that pH was one of the major drivers in the communities of saprotrophic fungi, and pH was positively correlated with the species richness of saprotrophic fungi in the range of pH 3.0–5.0. De et al. [72] also reported that litter saprotrophic fungi richness was positively correlated with soil pH in the range of pH 3.5–4.7. Yamanaka et al. [73] found that many of the saprotrophic species grew well around pH 7. It can be inferred from the above facts that saprotrophic fungi tend to flourish in a near-neutral pH environment, and too much acid or alkali will inhibit their growth. As we mentioned above, the largest difference between roofed and unroofed conditions is continuous rainfall, which was able to flush the remaining alkaline solution onto round bamboo. Therefore, compared with roofed bamboo samples, the surface of unroofed bamboo samples is a more suitable environment for saprotrophic fungi. Furthermore, the proportion of saprotrophs showed an increasing trend, and that of pathotrophs and symbiotrophs showed a declining trend in both roofed and unroofed groups. However, in our previous research, the proportion of saprotrophs showed a declining trend, whereas that of pathotrophs and symbiotrophs showed an increasing trend in roofed bamboo samples during deterioration [29]. We speculated that alkali treatment was the main reason for this difference. 

### 3.5. Chemical Composition Determination

**Figure 8:**
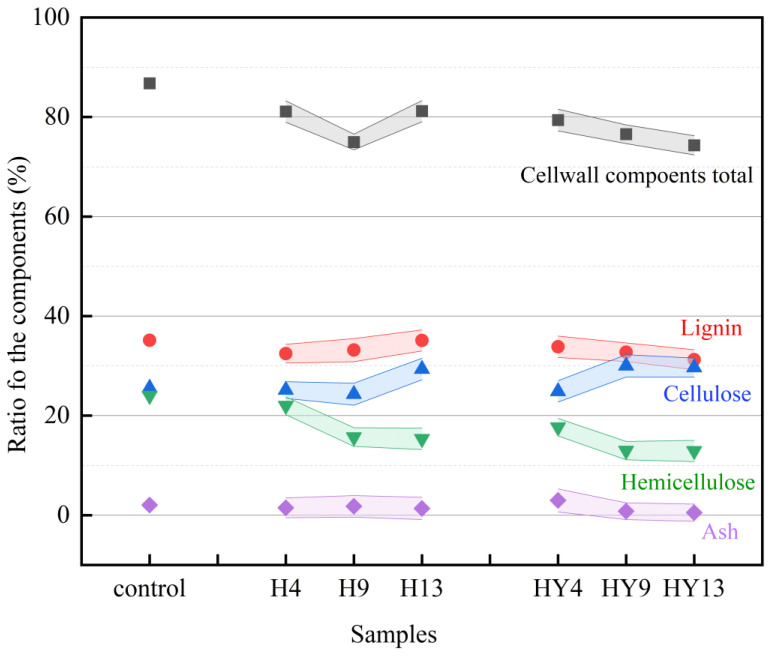
Changes in chemical composition of bamboo green (% *wt*/*wt*, dry weight basis).

Round bamboo was attacked by numerous microorganisms after being exposed to roofed and unroofed outdoor conditions for 4 to 13 weeks, which eventually affected its structure and properties. The surface of round bamboo, commercially known as bamboo green, is of great concern in round bamboo construction. Therefore, 1-mm-deep bamboo green was chosen for chemical composition analysis and property assessment. The relative amount of chemical composition, including lignin, hemicellulose, cellulose, and ash, is displayed in Figure 8. Research has shown that bamboo green differs from the inner culm in the proportion of the three major components, which accounts for a high ratio of lignin [74]. Bamboo green showed a decreasing amount of cell wall components total (CWT) in both roofed and unroofed circumstances, with unroofed bamboo exhibiting a CWT of up to 12.5%, suggesting a breakdown of bamboo cell wall from week 4 to week 13. 

The CWT amount of roofed and unroofed bamboo samples dropped by 5.7% and 7.4% at week_4, respectively, which is a relatively large reduction at the early stage of deterioration. In contrast to roofed bamboo samples, the CWT of unroofed groups showed a continuous decreasing trend, which mainly resulted from the declining ratio of lignin and cellulose. It has been reported in many studies that lignin is a kind of polyphenol that is sensitive to light, especially UV light [75]. Remarkably, the amount of hemicellulose in roofed and unroofed bamboo samples declined sharply by 8.3% and 11.1% at week_9, respectively, compared with the initial amount of hemicellulose in round bamboo samples, which may result from the growing relative abundance of Ascomycota. 

**Figure 9:**
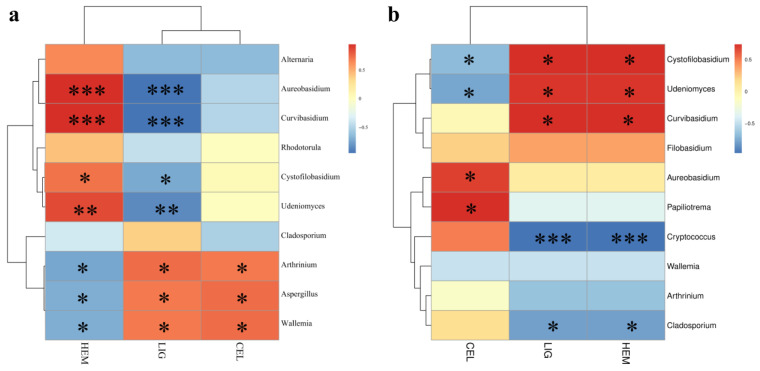
Spearman correlation heat map of the top ten genera and the relative content of hemicellulose, lignin, and cellulose of the roofed (**a**) and unroofed (**b**) groups. The colors in the heat maps indicate the Spearman correlation coefficient r, which ranges from −1 to 1; r < 0, negative correlation (blue); r > 0, positive correlation (red). Asterisks represent significance levels: * *p* < 0.05, ** *p* < 0.01, and *** *p* < 0.001. CEL: cellulose; LIG: lignin; HEM: hemicellulose.

A correlation analysis was conducted at the genus level between the fungal community and the relative contents of lignin, hemicellulose, and cellulose to figure out the factors contributing to the decomposition of bamboo (Figure 9). The relative content of lignin in roofed bamboo was negatively correlated with *Aureobasidium*, *Curvibasidium*, *Cystofilobasidium*, and *Udeniomyces*. The relative content of hemicellulose was negatively correlated with *Arthrinium*, *Aspergillus*, and *Wallemia*. *Aureobasidium* spp. are black, polyextremotolerant, and dimorphism yeast-like fungi with high biological and biotechnological importance [76]. They are capable of producing different types of extracellular enzymes for utilizing a wide range of substrates, including α-amylase for hydrolyzing starch [77], cellulase for utilizing cellulose [78], and laccase for degrading lignin [79]. Laccases (EC1.10.3.2, phenoloxidases) are multicopper oxidases with the ability to oxidize various organic and inorganic compounds, such as polyphenols, diphenols, and aromatic amines. Laccase is the only enzyme that can degrade lignin on its own and is widely distributed in fungi, bacteria, insects, and plants [80,81]. Fungal laccases have a high redox potential and play an extremely important role in lignin degradation. Hemicellulose is a major component of the complex biomass recalcitrance structure of fiber cell walls [82]. Unlike cellulose, hemicelluloses are heteropolysaccharides composed of xylose, arabinose, galactose, mannose, rhamnose, glucuronic acid, and galacturonic acid [83]. Bamboo hemicellulose is composed of more than 90% of xylan (4-O-acetyl-4-O-methyl-D-glucuronoxylan), a linear short-chain polymer with a polymerization degree of 200 [84]. Many species of the genus *Aspergillus* have been reported to produce xylanase, which is able to catalyze the hydrolysis of xylan, including *A. nidulans* [85], *A. niger* [86], *A. sulphureus* [87], *A. sydowii* [88], and *A. tubingensis* [89]. 

As to unroofed groups, the relative content of lignin and hemicellulose was negatively correlated with *Crytococcus* and *Cladosporium*. It’s noteworthy that the main microorganisms attributed to the degradation of cellulose in roofed and unroofed groups were different. In roofed groups, the content of cellulose was negatively correlated with *Alternaria*, *Aureobasidium*, *Curvibasidium*, and *Cladosporium*. Whereas, in unroofed groups, the content of cellulose was negatively correlated with *Cystofilobasidium*, *Udeniomyces*, and *Wellemia*. Chreptowicz et al. [90] also successfully isolated a strain of the genus *Cystofilobasidium*, named *C. macerans* WUT145, with extraordinarily high cellulolytic activity at 22 °C. This finding means that the genus *Cystofilobasidium* also has the potential to degrade cellulose. 

### 3.6. SEM Analysis

**Figure 10:**
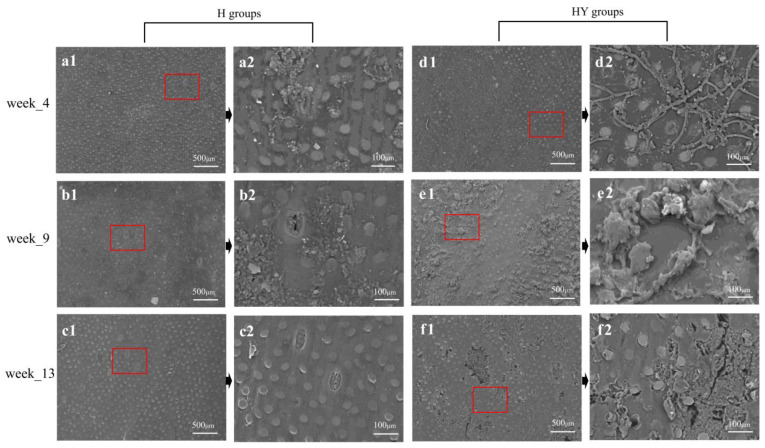
Scanning electron microscope (SEM) images of alkali-treated round bamboo samples in different deterioration stages. (**a1** and **a2**, **b1** and **b2**, **c1** and **c2**, **d1** and **d2**, **e1** and **e2**, and **f1** and **f2** are the pictures of the sample from H4, H9, H13, HY4, HY9, and HY13 groups, respectively). The red box is the original region of magnified picture.

Bamboo exposed to outdoor conditions experienced environmental and microbial influences, leading to changes in the chemical composition of green bamboo over time, resulting in alterations to its structure. The morphological characteristics of bamboo green were investigated using SEM, and the findings are illustrated in Figure 10. The surface structure of round bamboo continued to deteriorate from week 4 to week 13, with silicon cells that are compatible with the cortical tissue protruding and forming a distinct interface in their vicinity. Extensive hyphae can be found on the surface of unroofed round bamboo samples after 4 weeks of outdoor deterioration (Figure 10d2). Meanwhile, some hyphae start to penetrate the inner parts of bamboo via the silica stoma. After 9 weeks of deterioration, fungus hyphae tend to cluster together, forming small particles on the bamboo surface (Figure 10e2). The interface between silicon cells and the matrix became distinct after thirteen weeks of deterioration, and some cracks appeared on the surface. 

### 3.7. Contact Angle 

**Figure 11:**
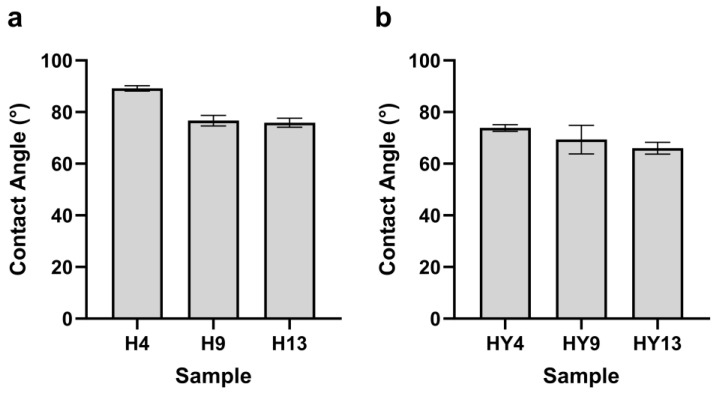
The contact angles of the bamboo green in the roofed (**a**) and unroofed (**b**) groups.

To study the change in hydrophobic property during deterioration, the contact angle of bamboo samples was measured. The contact angle of alkali-treated round bamboo in roofed and unroofed conditions was slightly lower than that of fresh round bamboo obtained from our previous study [29]. This phenomenon was also observed in a previous study [91]. The natural silica wax layer on the bamboo surface was able to prevent liquid from penetrating the bamboo. Soaking bamboo samples in an aqueous alkali solution is a straightforward and cost-effective way to remove the wax layer from the outer surfaces [92,93]. As shown in Figure 11, the contact angle of both roofed and unroofed samples fell dramatically during deterioration, suggesting a reduction in the hydrophobicity of round bamboo. As the three primary components, lignin is hydrophobic owing to aromatic rings [94], whereas cellulose and hemicellulose are hydrophilic due to numerous hydroxyl groups. The contact angles of bamboo culms were observed to decrease by 12% after the fungal biodegradation of lignin [95]. As demonstrated in Figure 8, the relative content of lignin in both roofed and unroofed groups decreased to some extent during outdoor degradation. Thus, we inferred that one of the causes contributing to the decrease in hydrophobicity in bamboo surfaces would be the reduction of lignin. Meanwhile, the constant exfoliation of the hydrophobic waxy layer may also affect the hydrophobicity of round bamboo. 

## 4. Conclusions 

This study shed light on the temporal dynamics of fungal communities in alkali-treated round bamboo outdoor deterioration under natural weathering. The diversity of fungal communities exhibited significant temporal variations, with Ascomycota and Basidiomycota being the dominant phyla in both roofed and unroofed environments throughout the deterioration period. *Curvibasidium* and *Cystofilobasidium* were the dominant genera in the initial stage but almost disappeared in the late stage of deterioration, which may be attributed to the rising temperature. The results of the RDA indicate that temperature was the most significant environmental factor contributing to the diversity and abundance of fungal communities. The content of cellulose, lignin, and hemicellulose decreased more or less during the deterioration. SEM and contact angle analysis revealed that the structure of the round bamboo surface suffered constant damage, which resulted from fungi and environmental factors. The comparison of fungal communities between fresh and alkali-treated round bamboo revealed that the succession mode of those two materials is different in fungal diversity, community composition, and trophic type. Taken together, this study provided a preliminary understanding of the temporal variation of the fungal community in the process of alkali-treated round bamboo outdoor deterioration. However, there are some limitations to this study, including study materials, sampling time, and research method. A more detailed investigation is needed to quantify the contribution of various biotic and abiotic factors to round bamboo outdoor deterioration.

## Data Availability

Data are contained within the article and Supplementary Materials.

## Supplementary Materials

The following supporting information can be downloaded at: https://www.mdpi.com/article/10.3390/microorganisms12050858/s1. Figure S1: C3.1 (**a**) and C3.2 (**b**) biologically hazardous conditions; Figure S2: The number of ASVs of fungal community in different samples; Figure S3: Rarefaction curves of the fungal community at different stages; Figure S4: Shannon index of the fungal community at different stages; Figure S5: The UPGMA figure of the fungal community at different stages.
