# Supplementary material for: Temporal Dynamics of Fungal Communities in Alkali-Treated Round Bamboo Deterioration under Natural Weathering
Source: Microorganisms. 2024 Apr 25;12(5):858. doi: 10.3390/microorganisms12050858 (PMC11124218; doi:10.3390/microorganisms12050858)
Supplement: Supplementary file 1 [file microorganisms-12-00858-s001.zip › microorganisms-2918926-supplementary.pdf]

Supplementary Materials:

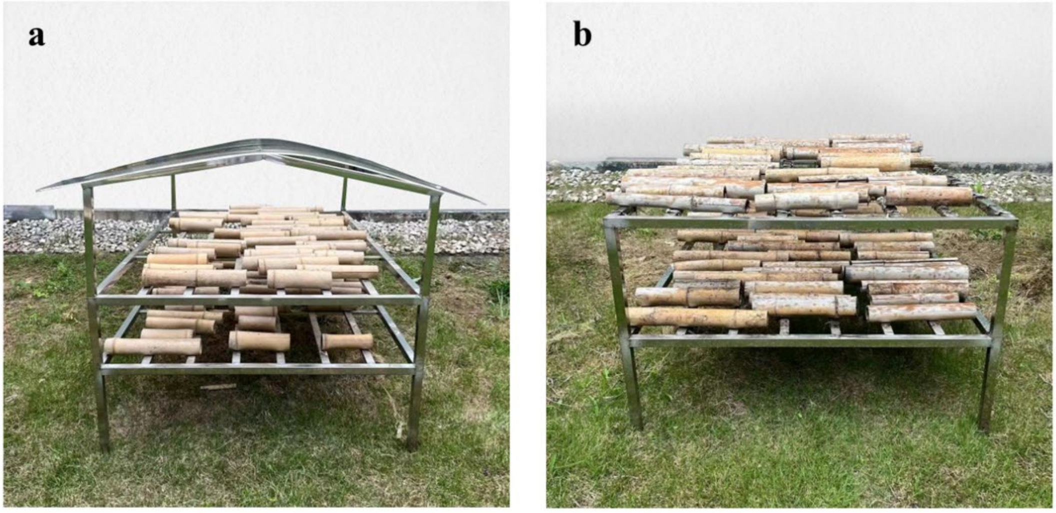

Figure S1. C3.1 (**a**) and C3.2 (**b**) biologically hazardous conditions.

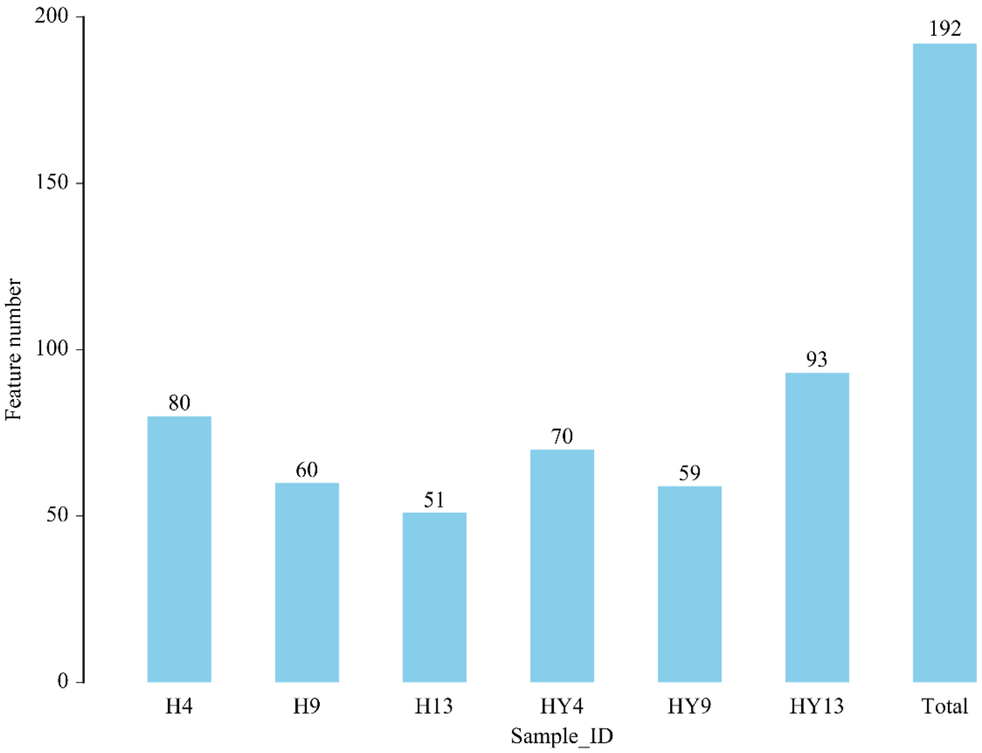

Figure S2. The number of ASVs of fungal community in different samples.

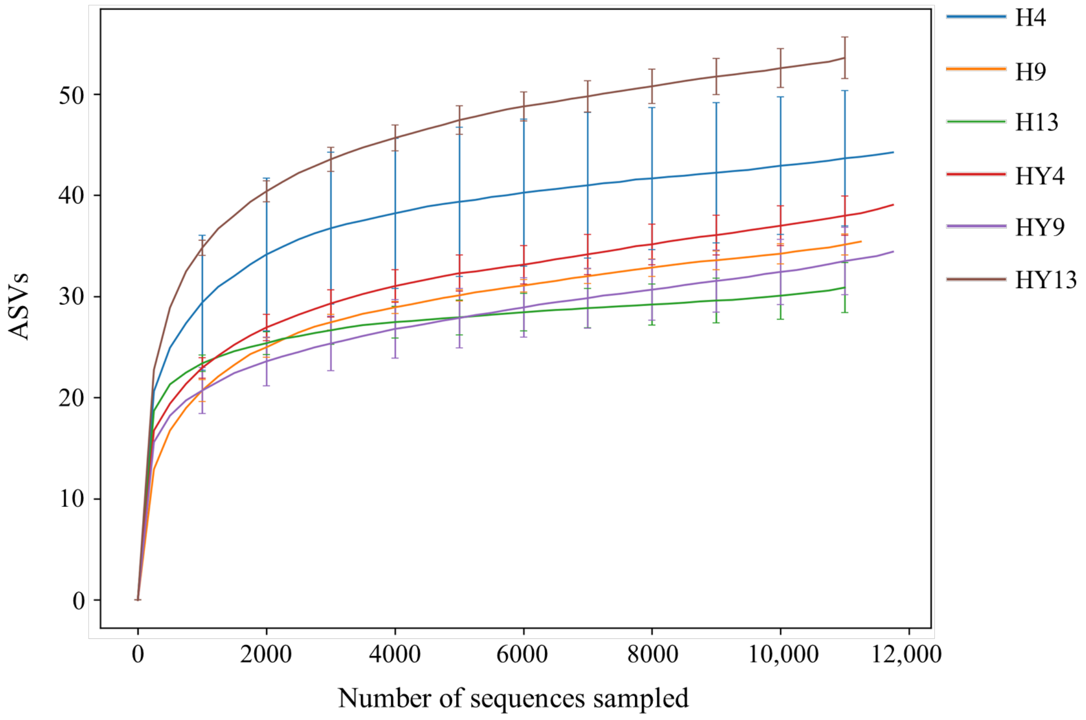

Figure S3. Rarefaction curves of the fungal community at different stages.

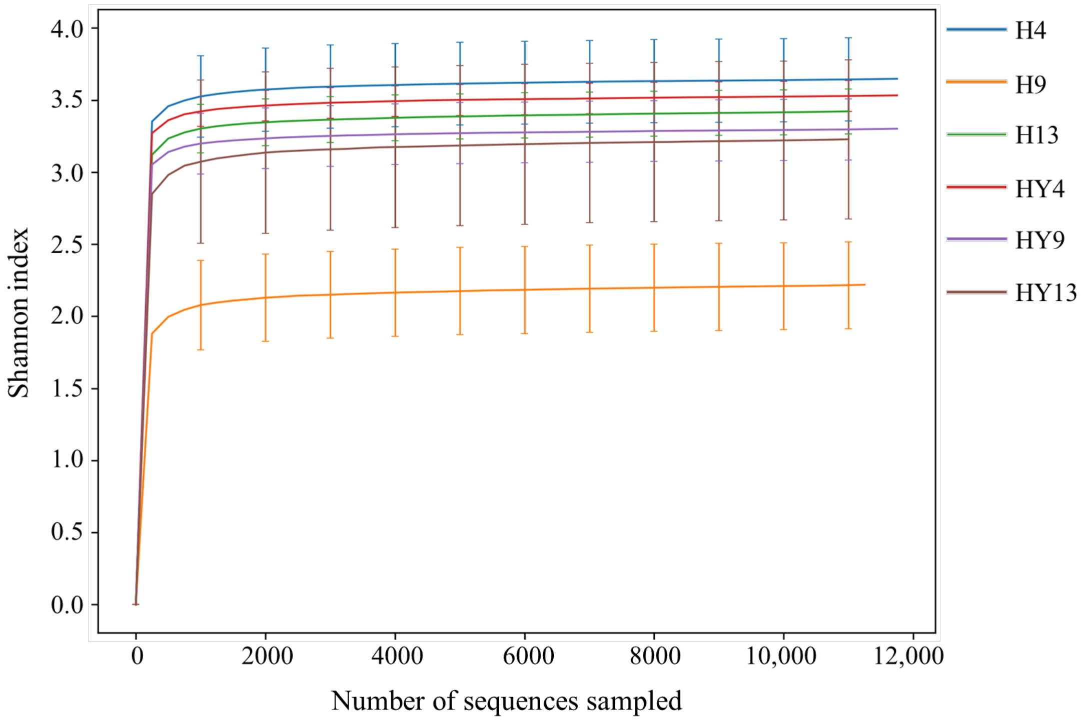

Figure S4. Shannon index of the fungal community at different stages.

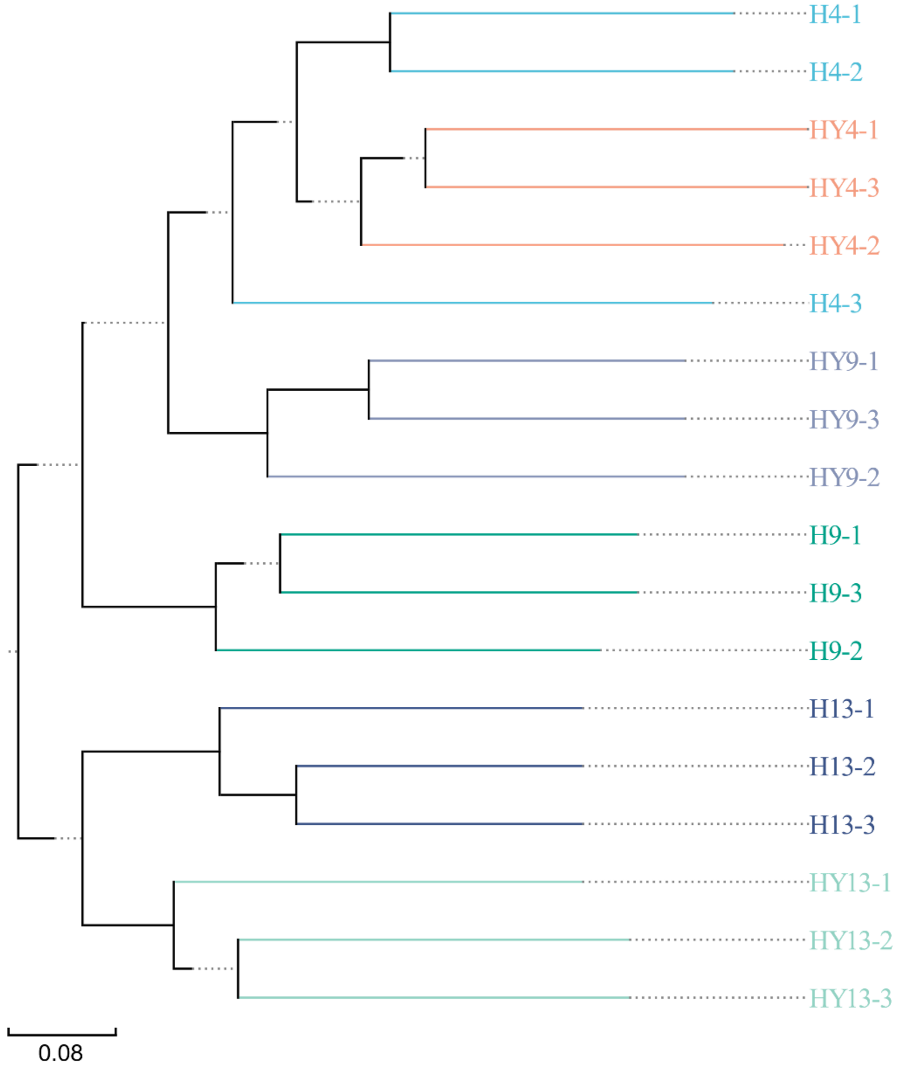

Figure S5. The UPGMA figure of the fungal community at different stages.
